# Supplementary material for: Splice-Junction-Based Mapping of Alternative Isoforms in the Human Proteome
Source: Cell Rep. Author manuscript; Available in PMC 2020 Jan 15. (PMC6961840; doi:10.1016/j.celrep.2019.11.026)
Supplement: 2 [file NIHMS1546469-supplement-2.zip › DF1/PXD006675/RightVentricle/RightVentricle_1_TMEM245_ESSEAVDRGESAPTLSTSPSPSSPSPTSPSPTLGR.pdf]

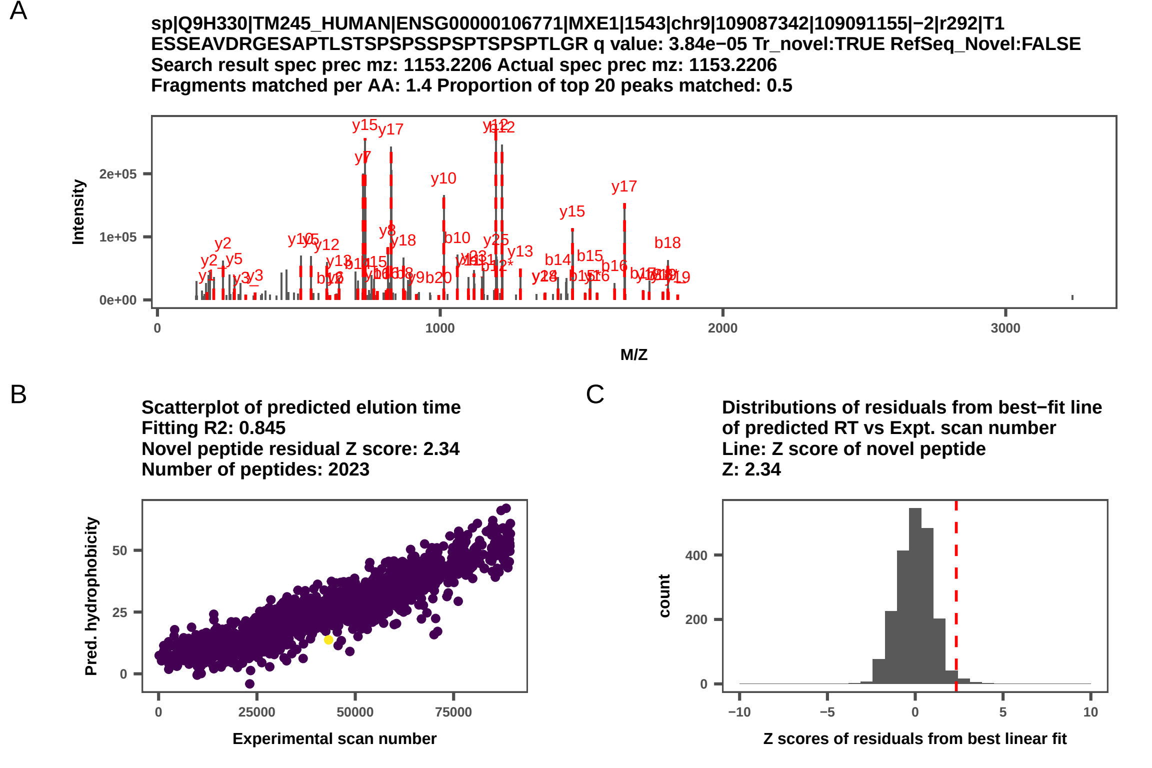
A
sp|Q9H330|TM245_HUMAN|ENSG00000106771|MXE1|1543|chr9|109087342|109091155|−2|r292|T1
ESSEAVDRGESAPTLSTSPSPSSPSPTSPSPTLGR q value: 3.84e−05 Tr_novel:TRUE RefSeq_Novel:FALSE
Search result spec prec mz: 1153.2206 Actual spec prec mz: 1153.2206
Fragments matched per AA: 1.4 Proportion of top 20 peaks matched: 0.5
Intensity
2e+05
1e+05
0e+00
0
1000
2000
3000
M/Z
y15
y17
y7
y10
y17
y15
b18
b10
b14
b15
b16
y2
y5
y13
y25
b20
B
Scatterplot of predicted elution time
Fitting R2: 0.845
Novel peptide residual Z score: 2.34
Number of peptides: 2023
Pred. hydrophobicity
50
25
0
0
25000
50000
75000
Experimental scan number
C
Distributions of residuals from best−fit line
of predicted RT vs Expt. scan number
Line: Z score of novel peptide
Z: 2.34
count
400
200
0
−10
−5
0
5
10
Z scores of residuals from best linear fit
